# Unraveling the complex role of microglia in Alzheimer’s disease: amyloid β metabolism and plaque formation

**DOI:** 10.1186/s41232-025-00383-4

**Published:** 2025-05-30

**Authors:** Sho Takatori, Mayuna Kondo, Taisuke Tomita

**Affiliations:** Laboratory of Neuropathology and Neuroscience, Graduate School of Pharmaceutical Sciences, The University of Tokyo, 7-3-1 Hongo, Bunkyo-ku Tokyo, 113-0033 Japan

**Keywords:** Alzheimer’s disease, Microglia, Amyloid-β, TREM2, Phagocytosis, Cerebral amyloid angiopathy, Glymphatic system

## Abstract

**Background:**

Alzheimer's disease (AD) is characterized by amyloid β (Aβ) accumulation in the brain. Recent genome-wide association studies have identified numerous AD risk genes highly expressed in microglia, highlighting their potential role in AD pathogenesis. Although microglia possess phagocytic capacity and have been implicated in Aβ clearance, accumulating evidence suggests their contribution to AD pathogenesis is more complex than initially anticipated.

**Main body:**

This review synthesizes current knowledge on microglial Aβ metabolism in AD, reconciling conflicting data from various studies. We examine evidence supporting the role of microglia in Aβ clearance, including studies on AD risk genes like TREM2 and their impact on microglial phagocytosis. Conversely, we explore findings that challenge this view, such as microglial depletion experiments resulting in unchanged or decreased Aβ accumulation. We propose that the contribution of microglia to Aβ metabolism is context-dependent, varying with disease progression, genetic background, and experimental conditions. Notably, microglia may promote parenchymal amyloid accumulation in early disease stages, while this accumulation-promoting effect may diminish in later stages. We discuss potential mechanisms for this paradoxical effect, including intracellular Aβ aggregation and release of pro-aggregation factors. Additionally, we explore the interplay between microglia-mediated Aβ metabolism and other clearance pathways, such as the glymphatic system, highlighting a potential compensatory relationship between parenchymal amyloid deposition and cerebral amyloid angiopathy.

**Conclusion:**

Our review underscores the complex and dynamic role of microglia in AD pathogenesis. Understanding the stage-specific functions of microglia in Aβ metabolism is crucial for developing targeted interventions. Future research should focus on elucidating the mechanisms of microglial functional changes throughout disease progression and determining the pathological significance of these changes. Exploring potential therapeutic strategies that selectively enhance beneficial microglial functions while mitigating their detrimental effects remains an important goal.

**Supplementary Information:**

The online version contains supplementary material available at 10.1186/s41232-025-00383-4.

## Introduction

Alzheimer’s disease (AD), the primary cause of dementia, is characterized by amyloid β (Aβ) accumulation in the brain, resulting from an imbalance between its production and clearance [1]. While familial AD is linked to mutations enhancing Aβ production, the mechanisms underlying sporadic AD remain unclear. Recent research suggests that impaired Aβ clearance, rather than overproduction, may be central to sporadic AD pathogenesis [2]. Understanding the mechanisms of impaired Aβ clearance in sporadic AD requires identifying key molecular players.

Microglia, the primary immune cells in the central nervous system (CNS), are essential for maintaining normal brain function. This is exemplified by hereditary diffuse leukoencephalopathy with spheroids (HDLS), caused by mutations in the macrophage colony-stimulating factor 1 receptor (*CSF1R*) gene [3]. HDLS leads to severe leukoencephalopathy through compromised microglial survival and function, underlining the pivotal role of these cells in CNS homeostasis. In line with this, recent genome-wide association studies have identified numerous AD risk genes highly expressed in microglia [4, 5]. Although their specific roles in AD pathogenesis remain incompletely elucidated, many of these genes appear to be involved in immune regulation (e.g., *CR1*, *CD33*, *PLCG2*), endocytosis/phagocytosis/membrane trafficking (*BIN1*, *PICALM*, *TREM2*, *ABI3*), or lipid metabolism (*APOE*, *CLU*, *ABCA7*, *INPP5D*) (For a comprehensive overview of these genes’ functions, readers are referred to previous reviews [6, 7]). Such genetic findings collectively point to a critical role for microglial function—or its dysfunction—in shaping AD pathogenesis.

Given their phagocytic capacity in the central nervous system, microglia were anticipated to contribute to the metabolism of extracellular Aβ deposits, thereby protecting against AD pathogenesis. However, recent findings present a more complex picture. While many studies demonstrate the ability of microglia to take up and degrade Aβ, others suggest their contribution might be limited or even lead to unexpected outcomes under certain conditions. Surprisingly, some microglial depletion experiments have shown no change or even a decrease in Aβ accumulation, challenging conventional views. Collectively, these contradictory observations imply that microglial function in AD is not monolithic but can change depending on disease stage or environmental cues.

Indeed, recent studies have revealed that microglia undergo dynamic state changes during AD progression. A single-cell transcriptomic study of AD mouse models identified a distinct microglial activation state termed disease-associated microglia (DAM), which is characterized by the suppression of homeostatic genes and upregulation of genes involved in lysosomal, phagocytic, and lipid metabolism pathways [8]. This finding has been further extended to human AD pathology by Sun et al., who identified 12 distinct microglial states, including AD-dysregulated homeostatic, inflammatory, and lipid-processing states [9]. These states show dynamic transitions during disease progression, suggesting that microglial function in AD is not static but rather evolves with disease advancement. The identification of these disease-specific microglial states has provided crucial insights into how these cells may contribute to AD pathogenesis at different stages.

This review aims to synthesize current knowledge on microglial Aβ metabolism and reconcile conflicting data. We propose that microglia may significantly influence Aβ metabolism under specific conditions but may not substantially contribute to steady-state Aβ clearance. We also explore the possibility that microglia might, in certain circumstances, promote brain parenchymal amyloid accumulation. Our discussion covers evidence supporting and challenging the role of microglia in Aβ metabolism, interprets conflicting results, explores mechanisms by which microglia might promote amyloid accumulation, and examines interactions between microglial-mediated Aβ metabolism and other clearance pathways. While the roles of microglia in AD extend beyond Aβ metabolism—including roles in amyloid plaque compaction and tau pathology—these aspects, though crucial, are beyond our scope and are covered extensively elsewhere [4, 7]. By examining the complex roles of microglia in Aβ metabolism, we aim to clarify their contribution to AD pathogenesis and identify promising avenues for future research and potential therapies.

## Microglial contribution to Aβ metabolism

### Is microglia truly involved in Aβ metabolism?

#### Evidence supporting the role of microglia in Aβ metabolism

Due to their phagocytic capabilities, microglia have long been considered crucial for Aβ metabolism^1^. Numerous studies provide evidence supporting the ability of microglia to metabolize Aβ and demonstrate that this ability can be enhanced under specific conditions.

Of particular interest is research on *Triggering Receptor Expressed on Myeloid Cells 2* (*TREM2*), a gene encoding a surface receptor essential for normal microglial function [10]. Biallelic loss-of-function mutations in *TREM2* or *TYROBP* (encoding its cytoplasmic adaptor molecule, also known as DAP12) cause Nasu-Hakola disease, also known as polycystic lipomembranous osteodysplasia with sclerosing leukoencephalopathy (PLOSL). This rare autosomal recessive disorder, characterized by early-onset frontotemporal dementia and multifocal bone cysts, underscores the crucial role of the TREM2–TYROBP pathway in maintaining microglial homeostasis. More recently, partial loss-of-function variants of *TREM2* have been identified as risk factors for AD, and a rare variant of *TYROBP* was likewise reported in early-onset AD [11], further highlighting the essential contribution of this receptor complex to disease pathogenesis. Indeed, multiple lines of evidence from Aβ-accumulation mouse models have shown that *Trem2* deficiency enhances Aβ pathology, although the timing and magnitude of this effect vary across different experimental settings [12–19] (See Additional file 1: Table S1 for a summary of TREM2 genetic intervention studies). In contrast, both human TREM2 transgenic mice [20] and Trem2 mutant mice with enhanced surface expression [21] showed reduced Aβ accumulation at specific disease stages. Conditional overexpression approaches further suggest that TREM2 can dynamically modulate Aβ accumulation depending on disease stage [22]. Collectively, these findings indicate that TREM2 plays a pivotal and context-dependent role in Aβ metabolism in AD.

TREM2 was initially found to regulate phagocytosis in microglia [23]. Subsequent studies using cultured microglia revealed its involvement in the uptake of Aβ aggregates [24, 25]. TREM2 directly recognizes Aβ oligomers [26] and fibrils [27] and indirectly promotes Aβ phagocytosis by regulating the expression of other Aβ receptors such as CD36 [28]. Downstream of TREM2, SYK is necessary for Aβ uptake [29, 30]. While TREM2 is crucial for microglial Aβ phagocytosis and metabolism, it is also deeply involved in mitigating amyloid toxicity through microglial clustering around amyloid plaques and promoting plaque compaction, which reduces the exposure of toxic Aβ species to surrounding neurons [13, 19, 31, 32]. Studies on TREM2 agonist antibodies have demonstrated their capacity to enhance microglial Aβ phagocytosis [33–35]. However, the therapeutic application of this approach has proven challenging, as exemplified by the recent failure of the TREM2-targeting monoclonal antibody AL002 in the phase 2 INVOKE-2 trial for early-stage AD (ClinicalTrials.gov identifier NCT04592874). The trial was discontinued after failing to meet its endpoints, with additional safety concerns arising from magnetic resonance imaging (MRI) changes resembling amyloid-related imaging abnormalities (ARIA). These clinical results underscore the complexity of translating promising preclinical findings into effective therapeutic strategies.

Other AD risk genes have also been associated with microglial Aβ metabolism. For instance, SPI1/PU.1, a transcription factor characterizing microglia, positively regulates Aβ phagocytosis [36, 37]. Conversely, the microglial receptor molecule CD33 negatively regulates Aβ uptake, partly by suppressing TREM2 downstream signaling [14]. Similarly, Leukocyte immunoglobulin-like receptor B4 (LILRB4), another inhibitory receptor, has been shown to regulate Aβ phagocytosis negatively [38]. Inositol polyphosphate-5-phosphatase D (INPP5D), which inhibits TREM2 downstream signaling, has also been suggested to negatively regulate microglial Aβ phagocytosis, although phenotypes vary across reports [39–43]. The cytoskeletal regulatory molecule ABI3 has also been reported to be involved in microglial Aβ metabolism [44–46].

Multiple scavenger receptors (macrophage scavenger receptor 1 (MSR1), CD36, scavenger receptor class B member 1 (SR-BI), receptor for advanced glycation end-product (RAGE)) have been identified as having Aβ binding capacity [47–52], although their role in in vivo Aβ metabolism remains debated [47, 53, 54]. Additionally, several membrane molecules that do not directly recognize Aβ are known to be involved in phagocytosis. The Tyro3, Axl, and Mer (TAM) receptor family members Axl and Mertk recognize phosphatidylserine co-deposited with amyloid plaques [55], while Piezo1, involved in mechanosensing, contributes to Aβ phagocytosis by recognizing the stiffness of amyloid plaques [56]. C–X3–C motif chemokine receptor 1 (CX3CR1) [57], C-type lectin domain containing 7 A (CLEC7A) [29, 30, 58], and G protein-coupled receptor 34 (GPR34) [59] also regulate microglial Aβ phagocytosis activity.

Furthermore, non-phagocytic Aβ uptake and degradation pathways are known. Notably, soluble Aβ is taken up by microglial macropinocytosis [60], and the possibility of Aβ degradation by microglia-derived secreted proteases has been suggested [61, 62].

Various immune and inflammatory signals can modulate microglial Aβ metabolism, either directly or indirectly through other cell types. For example, deficiency of inflammasome components such as NLR family pyrin domain containing 3 (NLRP3) or Caspase-1 increases microglial uptake of Aβ fibrils [63]. With Aβ deposition, microglia increase interleukin (IL)−3 receptor expression, and astrocyte-derived IL-3 promotes microglial Aβ metabolism [64]. IL-33 also promotes microglial Aβ metabolism, with the microglial Apolipoprotein E (ApoE)-vascular cell adhesion molecule 1 (VCAM1)-suppression of tumorigenicity 2 (ST2) axis playing a crucial role [65, 66]). Complement C3 or its receptor molecule CR3 deficiency decreases microglial Aβ phagocytosis [67].

Environmental factors also affect microglial Aβ metabolism. For example, the gut microbiome influences brain Aβ accumulation through microglial Aβ metabolism. Dodiya et al. showed that antibiotic administration to pre-weaning mice decreased Aβ deposition in a microglia-dependent manner [68]. Short-chain fatty acids derived from the gut microbiome have been reported to inhibit Aβ uptake by plaque-associated microglia [69]. Additionally, aging affects microglial Aβ phagocytic capacity [70, 71].

Lastly, microglial uptake of Aβ fibrils has been suggested to contribute to the mechanism of Aβ metabolism promotion by recently approved anti-Aβ antibody therapeutics [72].

**Fig. 1:**
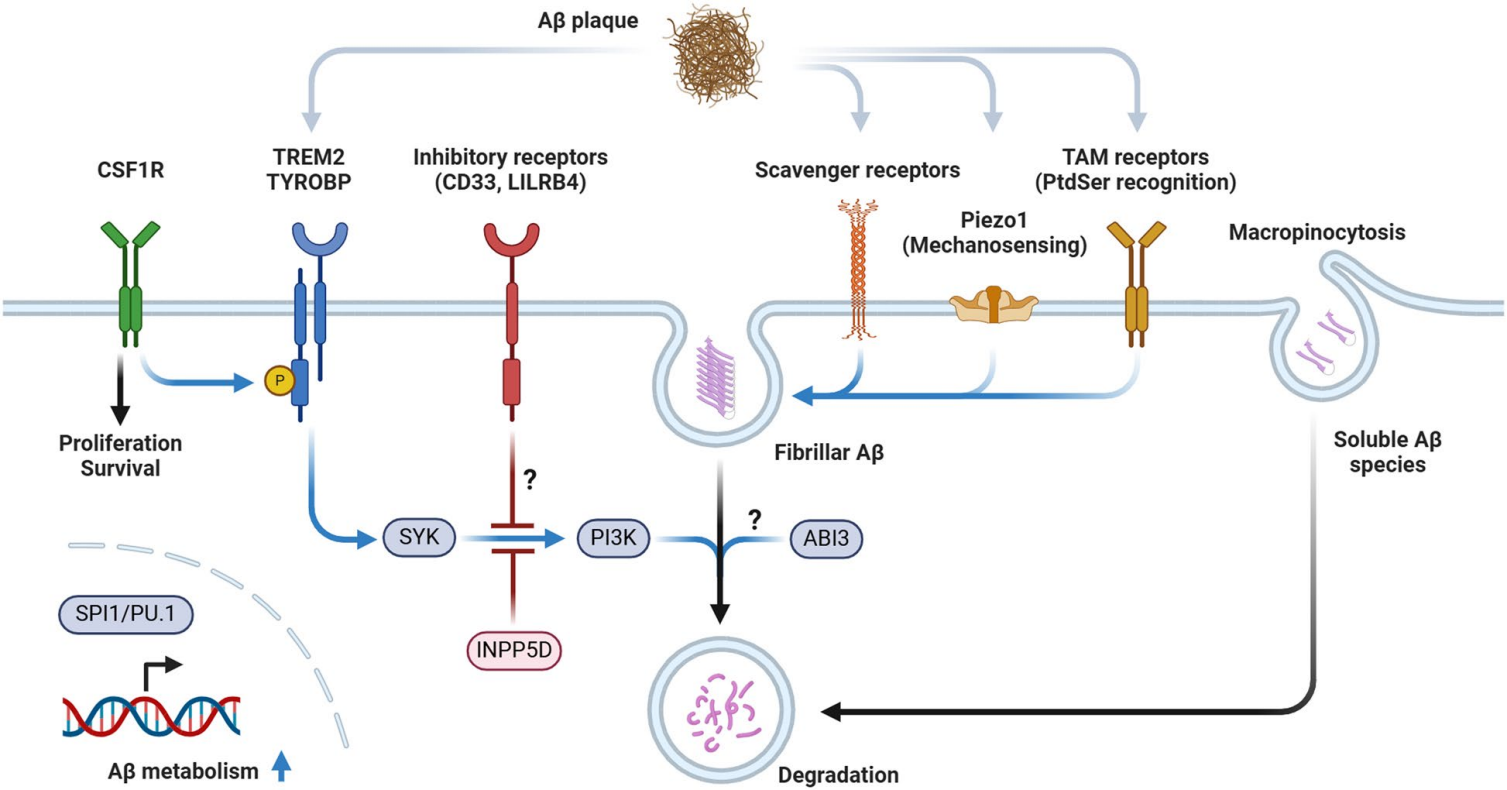
Molecular mechanisms involved in microglial Aβ uptake and degradation Schematic representation of pathways mediating microglial Aβ metabolism. Multiple receptors contribute to Aβ recognition and cellular responses: TREM2/TYROBP complex directly recognizes Aβ and triggers downstream signaling through SYK, CSF1R facilitates TREM2/TYROBP-mediated signaling through phosphorylation cascades while regulating microglial survival/proliferation, inhibitory receptors (CD33, LILRB4) negatively regulate Aβ uptake, scavenger receptors contribute to Aβ binding, Piezo1 senses plaque stiffness, and TAM receptors recognize phosphatidylserine co-deposited with amyloid plaques. Soluble Aβ species are internalized through macropinocytosis. These pathways converge on phosphatidylinositol 3-kinase (PI3K) signaling, which is modulated by INPP5D. The transcription factor SPI1/PU.1 regulates genes involved in Aβ metabolism. The fate of internalized Aβ, including whether and how efficiently it undergoes degradation, varies depending on the context. P: phosphorylation. Question marks indicate pathways that remain incompletely understood. Created with BioRender.com

These numerous findings collectively indicate that microglia play an important role in Aβ metabolism, though the specifics of this role may vary depending on the context and stage of disease progression (Fig. 1).

#### Evidence challenging the direct role of microglia in Aβ clearance

While substantial evidence supports the ability of microglia to uptake and degrade Aβ, conflicting reports also exist. Early histopathological studies already suggested potential limitations in microglial Aβ clearance, showing intact amyloid fibrils within microglial endosomes in AD patient brains and demonstrating the limited capacity of cultured microglia to degrade amyloid [73–75]. Recent genetic and pharmacological approaches have not only reinforced these observations but have also revealed an even more unexpected aspect of microglial function. Pharmacological microglial depletion experiments, primarily using CSF1R inhibitors, have yielded surprising results (see references [76, 77] for comprehensive summaries of microglial depletion studies). In many cases, Aβ accumulation remained essentially unchanged following microglial depletion [76, 78–87]. Even more intriguingly, some studies reported decreased parenchymal amyloid plaque formation upon microglial depletion. For instance, Spangenberg et al. found that administering the CSF1R inhibitor PLX3397 to the amyloid-laden 5xFAD mouse model reduced parenchymal amyloid plaque deposition, with treatment initiated as early as 1.5 months of age [85]. Similar results were obtained using a mouse model with deletion of the microglia-specific *Fms* intronic regulatory element in the *Csf1r* gene (FIRE^Δ/Δ^), which lacks microglia from birth [88].

These findings appear paradoxical at first glance. If microglia play a crucial role in Aβ metabolism, their removal should logically increase Aβ accumulation. However, the results suggest that microglia might not significantly contribute to steady-state Aβ metabolism or might even promote amyloid formation under certain conditions.

#### Interpretation of conflicting results

How should we interpret these conflicting results? While this contradiction has not been fully resolved, two important points need to be considered:

First, the results of microglial depletion experiments primarily reflect the contribution of microglia to steady-state Aβ metabolism. It is important to note that the lack of increased Aβ accumulation following microglial depletion does not rule out the Aβ metabolic capacity of microglia itself or the possibility of its enhancement under specific conditions.

**Fig. 2:**
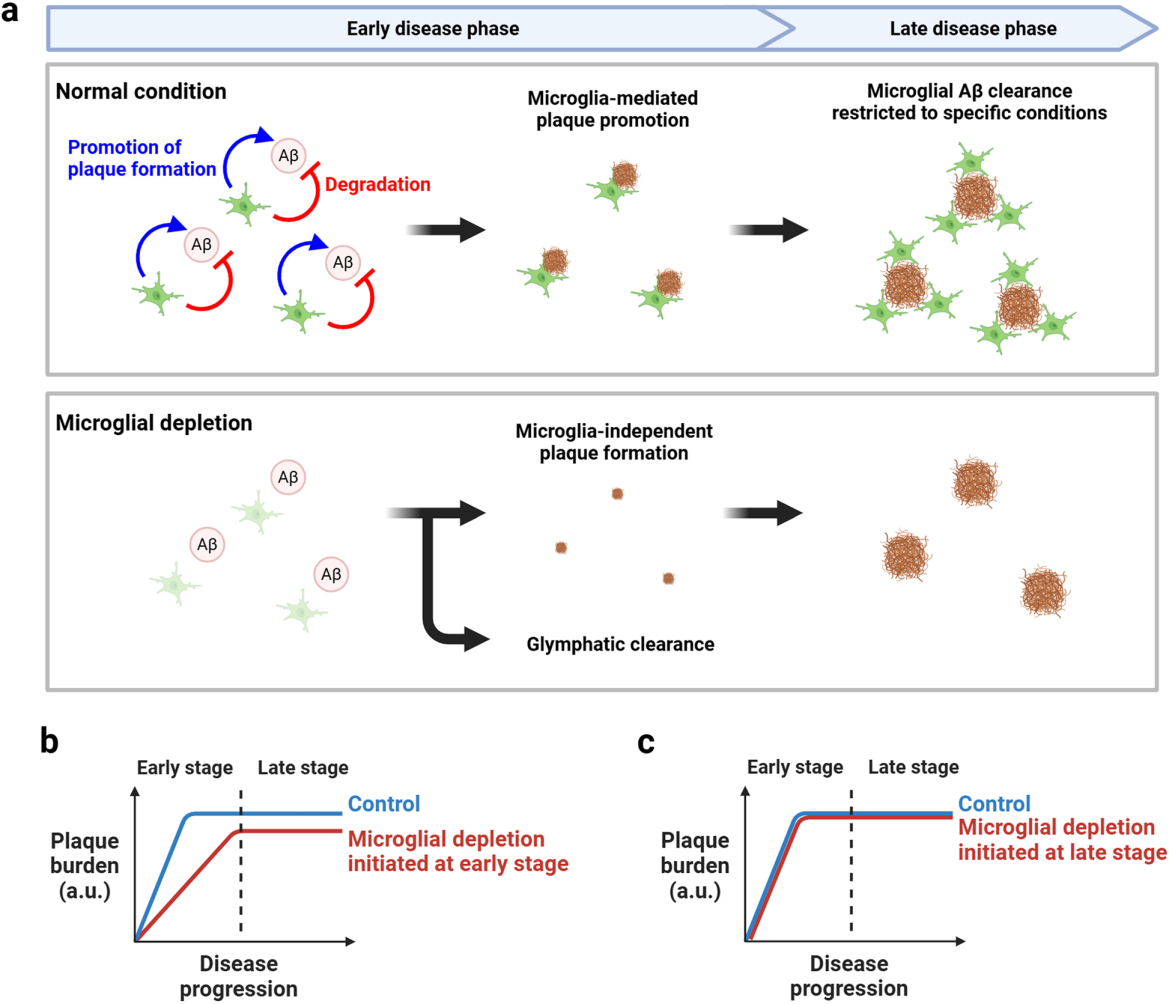
Stage-dependent roles of microglia in amyloid β accumulation **a** Schematic representation of microglial contribution to Aβ pathology progression. In normal condition (upper panel), microglia promote plaque formation during early disease phase while participating in Aβ metabolism only under specific conditions in late phase. In the absence of microglia (lower panel), plaque formation occurs in a microglia-independent manner with delayed kinetics, suggesting the involvement of alternative clearance pathways such as the glymphatic system. Green: microglia, Brown: amyloid plaque. **b**, **c** Schematic graphs showing how microglia depletion affects plaque burden when treatment is initiated in early (**b**) or late (**c**) disease stage. When microglia are depleted from early stage, plaque burden is reduced compared to control, reflecting removal of plaque-promoting effect by microglia (**b**). In contrast, when depletion is initiated at late stage, plaque burden remains largely unchanged (**c**), suggesting limited contribution of microglia to steady-state Aβ metabolism at this stage. Created with BioRender.com

Another crucial perspective is the timing of intervention. The results from Spangenberg et al. (2019) and Kiani Shabestari et al. (2022), as described in the previous section, are particularly noteworthy [85, 88]. In these studies, microglial depletion was initiated very early in the disease pathology. The observation of decreased Aβ accumulation in these cases suggests that the Aβ accumulation-promoting effect of microglia may be stage-specific. This hypothesis was directly tested by Baligács et al. (2024) [77]. Using the *App*^*NL−G−F*^ mouse model, they performed microglial depletion at different time points and found a biphasic result: depletion during the early accumulation phase (1–4 months of age) decreased Aβ accumulation, while depletion from 3 to 7 months of age showed no change in accumulation. Moreover, they demonstrated that transplanting human microglial cells into FIRE^Δ/Δ^ mice—which exhibited delayed amyloid plaque formation—significantly enhanced plaque formation. These results indicate that microglia may promote parenchymal Aβ accumulation primarily during the early stages of the disease, and as discussed later in Sect. 2.2, additional evidence further supports this notion. Synthesizing these observations, a new hypothesis emerges (Fig. 2).

1. Microglia exhibit stage-dependent effects on Aβ accumulation, promoting parenchymal Aβ deposition during early disease stages, while this accumulation-promoting effect diminishes as the disease progresses.
2. The Aβ metabolic capacity of microglia is limited under steady-state conditions but can be significantly enhanced under specific circumstances, such as genetic modifications, changes in the brain microenvironment, or targeted therapeutic interventions.

This hypothesis can uniformly explain the seemingly contradictory experimental results. In other words, while microglia indeed possess Aβ metabolic capacity, their role significantly changes depending on the stage of disease progression, genetic background, and environmental conditions. Further research should verify this hypothesis and elucidate the mechanisms of microglial functional changes.

### Mechanisms by which microglia promote parenchymal amyloid accumulation

As discussed in the previous section, microglia may promote Aβ accumulation, but the underlying mechanisms are not fully understood. Several in vitro experimental studies have provided important mechanistic insights into this phenomenon. For instance, Sheedy et al. (2013) demonstrated in experiments using bone marrow-derived macrophages that internalized soluble Aβ can transform into Thioflavin-positive amyloid within cells [89]. Moreover, Bassil et al. demonstrated that when human-induced pluripotent stem cell (iPSC)-derived microglia were added to a co-culture of neurons and astrocytes, and exposed to Aβ oligomers, they observed microglial uptake of Aβ and clustering of microglia around Aβ. Subsequently, amyloid plaque-like Aβ aggregates formed at these sites of microglial accumulation [90]. These results support the following hypothesis for the mechanism by which microglia promote Aβ accumulation: Microglia actively take up Aβ but cannot completely degrade it, leading to the accumulation of undegraded Aβ within cells. This concentrated Aβ may form amyloid, which could be released extracellularly due to cellular stress, serving as a seed for amyloid plaque formation.

In line with these in vitro observations, d’Errico et al. (2022) showed that transplantation of wild-type neural tissue into an AD model mouse led to infiltration of host-derived microglia, which in turn enhanced Aβ plaque formation at the graft site [91]. This direct demonstration of microglia promoting plaque accumulation in vivo further underscores their active role in driving parenchymal Aβ pathology. A recent study by Kaji et al. (2024) has revealed another important mechanism involving ApoE [92]. They demonstrated that ApoE can form fibrillar aggregates within the endo-lysosomal system of microglia, and these aggregates can trigger Aβ amyloidosis. This finding suggests that microglial uptake and processing of ApoE might serve as an initiating event in amyloid plaque formation.

Similarly, factors secreted by microglia may promote Aβ aggregation. Notably, Venegas et al. showed that microglia-derived apoptosis-associated speck-like protein containing a CARD (ASC) speck can cross-seed Aβ. In other words, ASC specks released by microglia as part of the inflammatory response may function as seeds for Aβ aggregation, potentially promoting amyloid plaque formation [93]. These mechanisms are not mutually exclusive and may act cooperatively at different stages of pathology or under various conditions.

## Crosstalk with other Aβ clearance pathways

The regulation of brain Aβ levels involves various clearance pathways. While the role of microglia in Aβ metabolism remains complex as discussed above, the glymphatic system represents another major clearance route from the brain [94]. Interestingly, results from microglial depletion experiments suggest a significant interplay between microglial function and glymphatic clearance.

Of particular note is that while microglial depletion can reduce Aβ accumulation in brain parenchyma, it often exacerbates cerebral amyloid angiopathy (CAA), characterized by Aβ deposition in cerebral blood vessel walls. This trade-off between parenchymal amyloid and CAA has been consistently reported in various experimental models. For instance, both Spangenberg et al. (2019) and Kiani Shabestari et al. (2022) observed this phenomenon in their microglial depletion studies, despite using different approaches (pharmacological and genetic, respectively) [85, 88]. Similar phenomena have been observed in other experimental models. For example, an increase of transforming growth factor-β1 (TGF-β1) production in human β-amyloid precursor protein-expressing mice reduced parenchymal amyloid plaques but increased amyloid accumulation in blood vessels [95]. Comparable results have been reported in ApoE4 knock-in 5xFAD mice [96] and Clusterin-deficient APP/PS1 mice [97]. These findings strongly suggest a compensatory relationship between amyloid deposition in brain parenchyma and blood vessel walls. When plaque formation in the brain parenchyma is suppressed, Aβ may be metabolized through perivascular pathways, potentially leading to CAA.

A related experimental observation comes from Feng et al. (2020) [98], who reported that while microglial depletion alone did not increase Aβ accumulation in APP/PS1 mice, it did so specifically when combined with deletion of *Aqp4*, a crucial component of the glymphatic system. This result further supports the hypothesis that microglia-mediated Aβ metabolism and glymphatic system-mediated Aβ clearance are complementary in regulating brain Aβ levels. Altogether, these findings indicate that microglia and other clearance pathways function in a complex interplay, where suppression of one route may redirect Aβ toward alternative pathways or alter its deposition pattern.

Importantly, this trade-off between parenchymal Aβ reduction and vascular deposition resembles the phenomenon observed in individuals receiving anti-Aβ antibody therapy, where clearing plaques can inadvertently exacerbate CAA, manifesting as ARIA [99]. ARIA can be severe or even fatal—particularly in patients with substantial preexisting CAA—and is characterized by local immune cell infiltration and antibody–Aβ complexes around cerebral vessels, collectively resembling CAA-related inflammation (CAA-ri). Although one hypothesis holds that high-affinity anti-Aβ antibodies might solubilize plaque-bound Aβ and redirect it to vascular sites [100], evidence for this “rerouting” mechanism is limited. By contrast, it appears more likely that the antibodies bind directly to Aβ already accumulated in vessel walls, triggering local inflammatory cascades [101]. In either scenario, the outcome partly parallels the microglial depletion model: reduced parenchymal amyloid but heightened vascular pathology. These parallels underscore the need for caution when manipulating microglial Aβ metabolism, as interventions that modulate one site of amyloid accumulation may worsen vascular amyloid pathology and increase the risk of ARIA.

## Conclusion

Our review has highlighted the complex and often paradoxical role of microglia in AD pathogenesis, particularly regarding Aβ metabolism. The apparent contradiction between microglial Aβ clearance capacity and their potential contribution to Aβ accumulation suggests a context-dependent function that changes during disease progression. This dynamic nature of microglial function presents both challenges and opportunities for therapeutic intervention. Several key questions emerge from our analysis that warrant further investigation:

1. Molecular mechanisms of plaque promotion: The precise mechanism by which microglia promote plaque formation—especially in the early stages of disease—remain unclear. Elucidating these mechanisms could reveal specific targets for therapeutic intervention aimed at inhibiting pathological plaque seeding.
2. Biological significance of microglial Aβ sequestration: It is still uncertain whether the sequestration of Aβ by microglia into parenchymal plaques is beneficial (e.g., isolating toxic Aβ species away from neurons [102]) or detrimental (e.g., by generating seeding sites for ongoing Aβ aggregation). Clarifying this is central to understanding how best to modulate microglial activity.
3. Factors controlling microglial transition: In this review, we have discussed how microglial roles in Aβ accumulation and metabolism change during disease progression. Identifying molecular and environmental factors that drive such functional transitions remains an essential challenge. Related to this point, as discussed in the “Introduction” section, microglia are known to undergo distinct state changes during disease progression, exemplified by DAM. Moreover, recent advances in spatial transcriptomics have revealed additional complexity by uncovering region-specific heterogeneity of microglial states in both AD patient brains and Aβ-accumulation model mice [103–106]. It will be intriguing to determine how these distinct microglial states correspond to the different functional states in Aβ accumulation and metabolism described in this review, which may provide crucial insights for developing stage- and region-specific therapeutic strategies.
4. Therapeutic targeting without broad depletion: From a therapeutic perspective, interventions that selectively modulate specific microglial functions—rather than broadly depleting or inhibiting microglia—are needed. For example, while CSF1R inhibitors may be an effective means to reduce unfavorable microglial populations, their indiscriminate action may risk recapitulating phenotypes seen in HDLS, which is caused by loss-of-function mutations in the *CSF1R* gene. Harnessing growing single-cell and spatial transcriptomic data could enable well-timed and highly specific approaches that address the heterogeneity of glial cell states.
5. Translating findings from mice to humans: Most evidence for microglial Aβ metabolism has been obtained in rodent models. Among the studies discussed in this review, a limited number have utilized human microglial cell cultures (e.g., references [36, 59, 90]), humanized knock-in mice (for molecules such as TREM2 [18, 20, 22] or LILRB4 [38]), or xenotransplantation of human microglia into AD mouse models (e.g., reference [77]), but such human-focused research remains relatively rare. Considering that transcriptomic and functional differences between mouse and human microglia are increasingly recognized, expanding these human-relevant approaches is critical. Future work employing humanized knock-in or xenograft models may be particularly useful for confirming or revising insights drawn from murine experiments. This will be essential not only for validating fundamental mechanisms but also for informing therapeutic strategies aimed at human-specific pathways of disease progression.

Addressing these questions will deepen our understanding of AD pathogenesis and may open new therapeutic opportunities. Continued exploration of region- and state-specific glial diversity can facilitate the development of tailored treatments that mitigate harmful microglial activities while preserving or enhancing neuroprotective roles. The key may lie in understanding and manipulating the context-dependent nature of microglia, paving the way toward more targeted and effective therapies for this devastating disease.

## Supplementary Information

Supplementary Material 1. Table S1. A summary of TREM2 genetic intervention studies organized by categories (TREM2 deficiency, upregulation, and loss-of-function (LOF) mutations). Different extraction methods were used for Aβ analysis: guanidine (Gdn), formic acid (FA), Triton X-100 (TX), phosphate-buffered saline (PBS), and Tris-buffered saline (TBS). The extracted fractions are abbreviated as"fxn". Changes in amyloid plaque burden and Aβ concentration compared to control conditions are indicated.

## Data Availability

Not applicable.

## Abbreviations

AD: Alzheimer 's disease
Aβ: Amyloid β
CNS: Central nervous system
HDLS: Hereditary diffuse leukoencephalopathy with spheroids
CSF1R: Macrophage colony-stimulating factor 1 receptor
DAM: Disease-associated microglia
TREM2: Triggering receptor expressed on myeloid cells 2
PLOSL: Polycystic lipomembranous osteodysplasia with sclerosing leukoencephalopathy
MRI: Magnetic resonance imaging
ARIA: Amyloid-related imaging abnormalities
LILRB4: Leukocyte immunoglobulin-like receptor B4
INPP5D: Inositol polyphosphate-5-phosphatase D
MSR1: Macrophage scavenger receptor 1
SR-BI: Scavenger receptor class B member 1
RAGE: Receptor for advanced glycation end-product
TAM receptor: Tyro3, Axl, and Mer receptor
CX3CR1: C-X3-C motif chemokine receptor 1
CLEC7A: C-type lectin domain containing 7A
GPR34: G protein–coupled receptor 34
NLRP3: NLR family pyrin domain containing 3
IL: Interleukin
ApoE: Apolipoprotein E
VCAM1: Vascular cell adhesion molecule 1
ST2: Suppression of tumorigenicity 2
iPSC: Induced pluripotent stem cell
ASC: Apoptosis-associated speck-like protein containing a CARD
CAA: Cerebral amyloid angiopathy
TGF-β1: Transforming growth factor-β1

## Footnotes

1 In this review, we employ “internalize” or “uptake” to describe instances where microglia incorporate Aβ, and we reserve “phagocytosis” for cases in which there is direct evidence (e.g., microscopy, biochemical assays) of a phagocytic mechanism. We use “degradation” to refer to the microglia-driven breakdown of Aβ, which has been most clearly demonstrated under in vitro conditions where lysosomal or enzymatic activities can be confirmed. In in vivo studies, however, it is often difficult to distinguish whether Aβ clearance relies on direct microglial degradation or alternative pathways such as the glymphatic system. Accordingly, we employ broader terms such as “metabolism” or “clearance” when discussing scenarios in which multiple routes may be involved.

## References

[1] Selkoe DJ, Hardy J. The amyloid hypothesis of Alzheimer’s disease at 25 years. EMBO Mol Med. 2016;8:595–608. 27025652 10.15252/emmm.201606210 PMC4888851

[2] Mawuenyega KG, Sigurdson W, Ovod V, Munsell L, Kasten T, Morris JC, et al. Decreased clearance of CNS beta-amyloid in Alzheimer’s disease. Science. 2010;330(6012):1774. 10.1126/science.1197623 PMC3073454 21148344

[3] Rademakers R, Baker M, Nicholson AM, Rutherford NJ, Finch N, Soto-Ortolaza A, et al. Mutations in the colony stimulating factor 1 receptor (CSF1R) gene cause hereditary diffuse leukoencephalopathy with spheroids. Nat Genet. 2011;44:200–5. 22197934 10.1038/ng.1027 PMC3267847

[4] Hansen DV, Hanson JE, Sheng M. Microglia in Alzheimer’s disease. J Cell Biol. 2018;217:459–72. 29196460 10.1083/jcb.201709069 PMC5800817

[5] Bellenguez C, Küçükali F, Jansen IE, Kleineidam L, Moreno-Grau S, Amin N, et al. New insights into the genetic etiology of Alzheimer’s disease and related dementias. Nat Genet. 2022;54:412–36. 35379992 10.1038/s41588-022-01024-z PMC9005347

[6] Karch CM, Goate AM. Alzheimer’s disease risk genes and mechanisms of disease pathogenesis. Biol Psychiatry. 2015;77:43–51. 24951455 10.1016/j.biopsych.2014.05.006 PMC4234692

[7] Takatori S, Wang W, Iguchi A, Tomita T. Genetic risk factors for alzheimer disease: emerging roles of microglia in disease pathomechanisms. Adv Exp Med Biol. 2019;1118:83–116. 30747419 10.1007/978-3-030-05542-4_5

[8] Keren-Shaul H, Spinrad A, Weiner A, Matcovitch-Natan O, Dvir-Szternfeld R, Ulland TK, et al. A unique microglia type associated with restricting development of Alzheimer’s disease. Cell. 2017;169:1276-1290.e17. 28602351 10.1016/j.cell.2017.05.018

[9] Sun N, Victor MB, Park YP, Xiong X, Scannail AN, Leary N, et al. Human microglial state dynamics in Alzheimer’s disease progression. Cell. 2023;186:4386-4403.e29. 37774678 10.1016/j.cell.2023.08.037 PMC10644954

[10] Colonna M. The biology of TREM receptors. Nat Rev Immunol. 2023;23:580–94. 36750615 10.1038/s41577-023-00837-1 PMC9904274

[11] Pottier C, Ravenscroft TA, Brown PH, Finch NA, Baker M, Parsons M, et al. Neurobiology of aging TYROBP genetic variants in early-onset Alzheimer’s disease. Neurobiol Aging. 2016;48:222.e9-222.e15. 27658901 10.1016/j.neurobiolaging.2016.07.028 PMC5159294

[12] Wang Y, Cella M, Mallinson K, Ulrich JD, Young KL, Robinette ML, et al. TREM2 lipid sensing sustains the microglial response in an Alzheimer’s disease model. Cell. 2015;160:1061–71. 25728668 10.1016/j.cell.2015.01.049 PMC4477963

[13] Wang Y, Ulland TK, Ulrich JD, Song W, Tzaferis JA, Hole JT, et al. TREM2-mediated early microglial response limits diffusion and toxicity of amyloid plaques. J Exp Med. 2016;213:667–75. 27091843 10.1084/jem.20151948 PMC4854736

[14] Griciuc A, Patel S, Federico AN, Choi SH, Innes BJ, Oram MK, et al. TREM2 acts downstream of CD33 in modulating microglial pathology in Alzheimer’s disease. Neuron. 2019;103:820-835.e7. 31301936 10.1016/j.neuron.2019.06.010 PMC6728215

[15] Jay TR, Miller CM, Cheng PJ, Graham LC, Bemiller S, Broihier ML, et al. TREM2 deficiency eliminates TREM2+ inflammatory macrophages and ameliorates pathology in Alzheimer’s disease mouse models. J Exp Med. 2015;212:287–95. 25732305 10.1084/jem.20142322 PMC4354365

[16] Jay TR, Hirsch AM, Broihier ML, Miller CM, Neilson LE, Ransohoff RM, Lamb BT, et al. Disease progression-dependent effects of TREM2 deficiency in a mouse model of Alzheimer’s disease. J Neurosci. 2017;37:637–47. 28100745 10.1523/JNEUROSCI.2110-16.2016 PMC5242410

[17] Parhizkar S, Arzberger T, Brendel M, Kleinberger G, Deussing M, Focke C, Nuscher B, et al. Loss of TREM2 function increases amyloid seeding but reduces plaque-associated ApoE. Nat Neurosci. 2019;22:191–204. 30617257 10.1038/s41593-018-0296-9 PMC6417433

[18] Leyns CEG, Gratuze M, Narasimhan S, Jain N, Koscal LJ, Jiang H, et al. TREM2 function impedes tau seeding in neuritic plaques. Nature Neurosci. 2019;22(8):1217–22. 31235932 10.1038/s41593-019-0433-0 PMC6660358

[19] Meilandt WJ, Ngu H, Gogineni A, Lalehzadeh G, Lee SH, Srinivasan K, Imperio J, et al. Trem2 deletion reduces late-stage amyloid plaque accumulation, elevates the Aβ42:Aβ40 ratio, and exacerbates axonal dystrophy and dendritic spine loss in the PS2APP Alzheimer’s mouse model. J Neurosci. 2020;40:1956–74. 31980586 10.1523/JNEUROSCI.1871-19.2019 PMC7046459

[20] Lee CYD, Daggett A, Gu X, Jiang LL, Langfelder P, Li X, et al. Elevated TREM2 gene dosage reprograms microglia responsivity and ameliorates pathological phenotypes in Alzheimer’s disease models. Neuron. 2018;97:1032-1048.e5. 29518357 10.1016/j.neuron.2018.02.002 PMC5927822

[21] Dhandapani R, Neri M, Bernhard M, Brzak I, Schweizer T, Rudin S, et al. Sustained Trem2 stabilization accelerates microglia heterogeneity and Aβ pathology in a mouse model of Alzheimer’s disease. Cell Rep. 2022;39:39. 10.1016/j.celrep.2022.110883 35649351

[22] Zhao N, Qiao W, Li F, Ren Y, Zheng J, Martens YA, et al. Elevating microglia TREM2 reduces amyloid seeding and suppresses disease-associated microglia. J Exp Med. 2022;219:e20212479. 36107206 10.1084/jem.20212479 PMC9481739

[23] Takahashi K, Prinz M, Stagi M, Chechneva O, Neumann H. TREM2-transduced myeloid precursors mediate nervous tissue debris clearance and facilitate recovery in an animal model of multiple sclerosis. PLoS Med. 2007;4: e124. 17425404 10.1371/journal.pmed.0040124 PMC1851623

[24] Kleinberger G, Yamanishi Y, Suárez-Calvet M, Czirr E, Lohmann E, Cuyvers E, et al. TREM2 mutations implicated in neurodegeneration impair cell surface transport and phagocytosis. Sci Transl Med. 2014;6:243ra86. 24990881 10.1126/scitranslmed.3009093

[25] Xiang X, Werner G, Bohrmann B, Liesz A, Mazaheri F, Capell A, et al. TREM2 deficiency reduces the efficacy of immunotherapeutic amyloid clearance. EMBO Mol Med. 2016;8:992–1004. 27402340 10.15252/emmm.201606370 PMC5009806

[26] Zhao Y, Wu X, Li X, Jiang LL, Gui X, Liu Y, Sun Y, et al. TREM2 is a receptor for β-amyloid that mediates microglial function. Neuron. 2018;97:1023-1031.e7. 29518356 10.1016/j.neuron.2018.01.031 PMC5889092

[27] Belsare KD, Wu H, Mondal D, Bond A, Castillo E, Jin J, et al. Soluble TREM2 inhibits secondary nucleation of Aβ fibrillization and enhances cellular uptake of fibrillar Aβ. Proc Natl Acad Sci U S A. 2022;119: e2114486119. 35082148 10.1073/pnas.2114486119 PMC8812518

[28] Kim S-M, Mun B-R, Lee S-J, Joh Y, Lee H-Y, Ji K-Y, et al. TREM2 promotes Aβ phagocytosis by upregulating C/EBPα-dependent CD36 expression in microglia. Sci Rep. 2017;7:11118. 28894284 10.1038/s41598-017-11634-x PMC5593901

[29] Ennerfelt H, Frost EL, Shapiro Daniel A, Holliday C, Zengeler KE, Voithofer G, Bolte AC, et al. SYK coordinates neuroprotective microglial responses in neurodegenerative disease. Cell. 2022;185:4135-4152.e22. 36257314 10.1016/j.cell.2022.09.030 PMC9617784

[30] Wang S, Sudan R, Peng V, Zhou Y, Du S, Yuede CM, et al. TREM2 drives microglia response to amyloid-β via SYK-dependent and -independent pathways. Cell. 2022;185:4153-4169.e19. 36306735 10.1016/j.cell.2022.09.033 PMC9625082

[31] Yuan P, Condello C, Keene CD, Wang Y, Bird TD, Paul SM, et al. TREM2 haplodeficiency in mice and humans impairs the microglia barrier function leading to decreased amyloid compaction and severe axonal dystrophy. Neuron. 2016;90:724–39. 27196974 10.1016/j.neuron.2016.05.003 PMC4898967

[32] Lee S-H, Meilandt WJ, Xie L, Gandham VD, Ngu H, Barck KH, et al. Trem2 restrains the enhancement of tau accumulation and neurodegeneration by β-amyloid pathology. Neuron. 2021;109:1283-1301.e6. 33675684 10.1016/j.neuron.2021.02.010

[33] Wang S, Mustafa M, Yuede CM, Salazar SV, Kong P, Long H, et al. Anti-human TREM2 induces microglia proliferation and reduces pathology in an Alzheimer’s disease model. J Exp Med. 2020;217:e20200785. 32579671 10.1084/jem.20200785 PMC7478730

[34] Price BR, Sudduth TL, Weekman Erica M, Johnson S, Hawthorne D, Woolums A, Wilcock DM. Therapeutic Trem2 activation ameliorates amyloid-beta deposition and improves cognition in the 5XFAD model of amyloid deposition. J Neuroinflammation. 2020;17:238. 32795308 10.1186/s12974-020-01915-0 PMC7427742

[35] Schlepckow K, Monroe KM, Kleinberger G, Cantuti-Castelvetri L, Parhizkar S, Xia D, Willem M, et al. Enhancing protective microglial activities with a dual function TREM2 antibody to the stalk region. EMBO Mol Med. 2020;12:e11227. 32154671 10.15252/emmm.201911227 PMC7136959

[36] Smith AM, Gibbons HM, Oldfield RL, Bergin PM, Mee EW, Faull RLM, et al. The transcription factor PU.1 is critical for viability and function of human brain microglia. Glia. 2013;61:929–42. 23483680 10.1002/glia.22486

[37] Huang KL, The International Genomics of Alzheimer’s Project, Marcora E, Pimenova AA, Di Narzo AF, Kapoor M, et al. A common haplotype lowers PU.1 expression in myeloid cells and delays onset of Alzheimer’s disease. Nat Neurosci. 2017;20:1052–61. 10.1038/nn.4587 PMC5759334 28628103

[38] Hou J, Chen Y, Cai Z, Seong Heo Gyu, Yuede CM, Wang Z, Lin K, et al. Antibody-mediated targeting of human microglial leukocyte Ig-like receptor B4 attenuates amyloid pathology in a mouse model. Sci Transl Med. 2024;16:eadj9052. 38569016 10.1126/scitranslmed.adj9052 PMC11977387

[39] Lin PB-C, Tsai AP-Y, Soni D, Lee-Gosselin A, Moutinho M, Puntambekar SS, et al. INPP5D deficiency attenuates amyloid pathology in a mouse model of Alzheimer’s disease. Alzheimers Dement. 2023;19:2528–37. 36524682 10.1002/alz.12849

[40] Iguchi A, Takatori S, Kimura S, Muneto H, Wang K, Etani H, et al. INPP5D modulates TREM2 loss-of-function phenotypes in a β-amyloidosis mouse model. iScience. 2023;26:106375. 37035000 10.1016/j.isci.2023.106375 PMC10074152

[41] Castranio EL, Hasel P, Haure-Mirande JV, Ramirez Jimenez AV, Hamilton BW, Kim RD, Glabe CG, et al. Microglial INPP5D limits plaque formation and glial reactivity in the PSAPP mouse model of Alzheimer’s disease. Alzheimers Dement. 2023;19:2239–52. 36448627 10.1002/alz.12821 PMC10481344

[42] Samuels JD, Moore KA, Ennerfelt Hannah E, Johnson AM, Walsh AE, Price Richard J, Lukens JR. The Alzheimer’s disease risk factor INPP5D restricts neuroprotective microglial responses in amyloid beta-mediated pathology. Alzheimers Dement. 2023;19:4908–21. 37061460 10.1002/alz.13089 PMC10576836

[43] Yin Z, Rosenzweig N, Kleemann KL, Zhang X, Brandão W, Margeta Milica A, Schroeder C, et al. APOE4 impairs the microglial response in Alzheimer’s disease by inducing TGFβ-mediated checkpoints. Nat Immunol. 2023;24:1839–53. 37749326 10.1038/s41590-023-01627-6 PMC10863749

[44] Karahan H, Smith DC, Kim B, Dabin LC, Al-Amin MM, Wijeratne HRS, et al. Deletion of Abi3 gene locus exacerbates neuropathological features of Alzheimer’s disease in a mouse model of Aβ amyloidosis. Sci Adv. 2021;7:eabe3954. 34731000 10.1126/sciadv.abe3954 PMC8565913

[45] Karahan H, Smith DC, Kim B, McCord B, Mantor J, John SK, et al. The effect of Abi3 locus deletion on the progression of Alzheimer’s disease-related pathologies. Front Immunol. 2023;14: 1102530. 36895556 10.3389/fimmu.2023.1102530 PMC9988916

[46] Ibanez KR, McFarland KN, Phillips J, Allen M, Lessard CB, Zobel L, De La Cruz EG, et al. Deletion of Abi3/Gngt2 influences age-progressive amyloid β and tau pathologies in distinctive ways. Alzheimer’s Res Ther. 2022;14:104. 35897046 10.1186/s13195-022-01044-1 PMC9327202

[47] El Khoury J, Hickman SE, Thomas CA, Cao L, Silverstein SC, Loike JD. Scavenger receptor-mediated adhesion of microglia to beta-amyloid fibrils. Nature. 1996;382:716–9. 8751442 10.1038/382716a0

[48] Yan SD, Chen X, Fu J, Chen M, Zhu H, Roher A, et al. RAGE and amyloid-beta peptide neurotoxicity in Alzheimer’s disease. Nature. 1996;382:685–91. 8751438 10.1038/382685a0

[49] Coraci IS, Husemann J, Berman JW, Hulette C, Dufour JH, Campanella GK, et al. CD36, a class B scavenger receptor, is expressed on microglia in Alzheimer’s disease brains and can mediate production of reactive oxygen species in response to β-amyloid fibrils. Am J Pathol. 2002;160:101–12. 11786404 10.1016/s0002-9440(10)64354-4 PMC1867121

[50] Bamberger ME, Harris ME, McDonald Douglas R, Husemann J, Landreth GE. A cell surface receptor complex for fibrillar β-amyloid mediates microglial activation. J Neurosci. 2003;23:2665–74. 12684452 10.1523/JNEUROSCI.23-07-02665.2003 PMC6742111

[51] El Khoury JB, Moore KJ, Means TK, Leung J, Terada K, Toft M, et al. CD36 mediates the innate host response to beta-amyloid. J Exp Med. 2003;197:1657–66. 12796468 10.1084/jem.20021546 PMC2193948

[52] Thanopoulou K, Fragkouli A, Stylianopoulou F, Georgopoulos S. Scavenger receptor class B type I (SR-BI) regulates perivascular macrophages and modifies amyloid pathology in an Alzheimer’s mouse model. Proc Natl Acad Sci U S A. 2010;107:20816–21. 21076037 10.1073/pnas.1005888107 PMC2996412

[53] Frenkel D, Wilkinson K, Zhao L, Hickman SE, Means TK, Puckett L, et al. Scara1 deficiency impairs clearance of soluble amyloid-β by mononuclear phagocytes and accelerates Alzheimer’s-like disease progression. Nat Commun. 2013;4:2030. 23799536 10.1038/ncomms3030 PMC3702268

[54] Huang F, Buttini M, Wyss-Coray T, McConlogue L, Kodama T, Pitas RE, et al. Elimination of the class A scavenger receptor does not affect amyloid plaque formation or neurodegeneration in transgenic mice expressing human amyloid protein precursors. Am J Pathol. 1999;155:1741–7. 10550330 10.1016/S0002-9440(10)65489-2 PMC1866996

[55] Huang Y, Happonen KE, Burrola PG, O’Connor C, Hah N, Huang L, et al. Microglia use TAM receptors to detect and engulf amyloid β plaques. Nat Immunol. 2021;22:586–94. 33859405 10.1038/s41590-021-00913-5 PMC8102389

[56] Hu J, Chen Q, Zhu H, Hou L, Liu W, Yang Q, et al. Microglial Piezo1 senses Aβ fibril stiffness to restrict Alzheimer’s disease. Neuron. 2023;111:15-29.e8. 36368316 10.1016/j.neuron.2022.10.021

[57] Liu Z, Condello C, Schain A, Harb R, Grutzendler J. CX3CR1 in microglia regulates brain amyloid deposition through selective protofibrillar amyloid-β phagocytosis. J Neurosci. 2010;30:17091–101. 21159979 10.1523/JNEUROSCI.4403-10.2010 PMC3077120

[58] Ennerfelt H, Holliday C, Shapiro DA, Zengeler KE, Bolte AC, Ulland TK, et al. CARD9 attenuates Aβ pathology and modifies microglial responses in an Alzheimer’s disease mouse model. Proc Natl Acad Sci U S A. 2023;120: e2303760120. 37276426 10.1073/pnas.2303760120 PMC10268238

[59] Etani H, Takatori S, Wang W, Omi J, Akahori A, Watanabe H, et al. Selective agonism of GPR34 stimulates microglial uptake and clearance of amyloid β fibrils. bioRxiv. 2024. 10.1101/2024.05.08.593262. 10.1186/s13195-025-01891-8 PMC12632051 41261421

[60] Mandrekar S, Jiang Q, Lee CYD, Koenigsknecht-Talboo J, Holtzman DM, Landreth GE. Microglia mediate the clearance of soluble Abeta through fluid phase macropinocytosis. J Neurosci. 2009;29:4252–62. 19339619 10.1523/JNEUROSCI.5572-08.2009 PMC3034143

[61] Qiu WQ, Ye Z, Kholodenko D, Seubert P, Selkoe DJ. Degradation of amyloid β-protein by a metalloprotease secreted by microglia and other neural and non-neural cells. J Biol Chem. 1997;272:6641–6. 9045694 10.1074/jbc.272.10.6641

[62] Tamboli IY, Barth E, Christian L, Siepmann M, Kumar S, Singh S, et al. Statins promote the degradation of extracellular amyloid β-peptide by microglia via stimulation of exosome-associated insulin-degrading enzyme (IDE) secretion. J Biol Chem. 2010;285:37405–14. 20876579 10.1074/jbc.M110.149468 PMC2988346

[63] Heneka MT, Kummer MP, Stutz A, Delekate A, Schwartz S, Vieira-Saecker A, et al. NLRP3 is activated in Alzheimer’s disease and contributes to pathology in APP/PS1 mice. Nature. 2013;493:674–8. 23254930 10.1038/nature11729 PMC3812809

[64] McAlpine CS, Park J, Griciuc A, Kim E, Choi SH, Iwamoto Y, et al. Astrocytic interleukin-3 programs microglia and limits Alzheimer’s disease. Nature. 2021;595:701–6. 34262178 10.1038/s41586-021-03734-6 PMC8934148

[65] He D, Xu H, Zhang H, Tang R, Lan Y, Xing R, et al. Disruption of the IL-33-ST2-AKT signaling axis impairs neurodevelopment by inhibiting microglial metabolic adaptation and phagocytic function. Immunity. 2022;55:159-173.e9. 34982959 10.1016/j.immuni.2021.12.001 PMC9074730

[66] Lau S-F, Wu W, Wong HY, Ouyang L, Qiao Y, Xu J, et al. The VCAM1-ApoE pathway directs microglial chemotaxis and alleviates Alzheimer’s disease pathology. Nat Aging. 2023;3:1219–36. 37735240 10.1038/s43587-023-00491-1 PMC10570140

[67] Fu H, Liu B, Frost JL, Hong S, Jin M, Ostaszewski B, et al. Complement component C3 and complement receptor type 3 contribute to the phagocytosis and clearance of fibrillar Aβ by microglia. Glia. 2012;60:993–1003. 22438044 10.1002/glia.22331 PMC3325361

[68] Dodiya HB, Lutz HL, Weigle IQ, Patel P, Michalkiewicz J, Roman-Santiago CJ, et al. Gut microbiota-driven brain Aβ amyloidosis in mice requires microglia. J Exp Med. 2022;219:e20200895. 34854884 10.1084/jem.20200895 PMC8647415

[69] Colombo AV, Sadler RK, Llovera G, Singh V, Roth S, Heindl S, et al. Microbiota-derived short chain fatty acids modulate microglia and promote Aβ plaque deposition. Elife. 2021;10:10. 10.7554/eLife.59826 PMC8043748 33845942

[70] Pluvinage J V, Haney MS, Smith Benjamin A H and Sun J, Iram T, Bonanno L, Li Lulin and Lee DP, et al. CD22 blockade restores homeostatic microglial phagocytosis in ageing brains. Nature. 2019;568:187–92. 30944478 10.1038/s41586-019-1088-4 PMC6574119

[71] Thomas AL, Lehn MA, Janssen EM, Hildeman DA, Chougnet CA. Naturally-aged microglia exhibit phagocytic dysfunction accompanied by gene expression changes reflective of underlying neurologic disease. Sci Rep. 2022;12:19471. 36376530 10.1038/s41598-022-21920-y PMC9663419

[72] Loeffler DA. Antibody-mediated clearance of brain amyloid-β: mechanisms of action, effects of natural and monoclonal anti-Aβ antibodies, and downstream effects. J Alzheimers Dis Rep. 2023;7:873–99. 37662616 10.3233/ADR-230025 PMC10473157

[73] Wisniewski HM, Vorbrodt AW, Wegiel J, Morys J, Lossinsky AS. Ultrastructure of the cells forming amyloid fibers in Alzheimer disease and scrapie. Am J Med Genet Suppl. 1990;7:287–97. 1963537 10.1002/ajmg.1320370757

[74] Frackowiak J, Wisniewski HM, Wegiel J, Merz GS, Iqbal K, Wang KC. Ultrastructure of the microglia that phagocytose amyloid and the microglia that produce ?-amyloid fibrils. Acta Neuropathol. 1992;84:225. 1414275 10.1007/BF00227813

[75] Paresce DM, Chung H, Maxfield FR. Slow degradation of aggregates of the Alzheimer’s disease amyloid β-protein by microglial cells. J Biol Chem. 1997;272:29390–7. 9361021 10.1074/jbc.272.46.29390

[76] Clayton K, Delpech JC, Herron S, Iwahara N, Ericsson M, Saito T, Saido TC, et al. Plaque associated microglia hyper-secrete extracellular vesicles and accelerate tau propagation in a humanized APP mouse model. Mol Neurodegener. 2021;16:18. 33752701 10.1186/s13024-021-00440-9 PMC7986521

[77] Baligács N, Albertini G, Borrie SC, Serneels L, Pridans C, Balusu S, et al. Homeostatic microglia initially seed and activated microglia later reshape amyloid plaques in Alzheimer’s Disease. Nat Commun. 2024;15:10634. 39639016 10.1038/s41467-024-54779-w PMC11621353

[78] Casali BT, MacPherson KP, Reed-Geaghan EG, Landreth GE. Microglia depletion rapidly and reversibly alters amyloid pathology by modification of plaque compaction and morphologies. Neurobiol Dis. 2020;142: 104956. 32479996 10.1016/j.nbd.2020.104956 PMC7526856

[79] Dagher NN, Najafi AR, Kayala KM, Elmore MRP, White TE, Medeiros R, West BL, Green KN. Colony-stimulating factor 1 receptor inhibition prevents microglial plaque association and improves cognition in 3xTg-AD mice. J Neuroinflammation. 2015;12:139. 26232154 10.1186/s12974-015-0366-9 PMC4522109

[80] Grathwohl SA, Kälin RE, Bolmont T, Prokop S, Winkelmann G, Kaeser SA, Odenthal J, Radde R, et al. Formation and maintenance of Alzheimer’s disease beta-amyloid plaques in the absence of microglia. Nat Neurosci. 2009;12:1361–3. 19838177 10.1038/nn.2432 PMC4721582

[81] Olmos-Alonso A, Schetters STT, Sarmi Sri, Askew K, Mancuso R, Vargas-Caballero M, Holscher C, et al. Pharmacological targeting of CSF1R inhibits microglial proliferation and prevents the progression of Alzheimer’s-like pathology. Brain. 2016;139(Pt 3):891–907. 26747862 10.1093/brain/awv379 PMC4766375

[82] Prokop S, Miller KR, Drost N, Handrick S, Mathur V, Luo J, et al. Impact of peripheral myeloid cells on amyloid-β pathology in Alzheimer’s disease-like mice. J Exp Med. 2015;212:1811–8. 26458768 10.1084/jem.20150479 PMC4612091

[83] Son Y, Jeong YJ, Shin NR, Jong Oh Se, Nam KR, Choi HD, Choi JY, et al. Inhibition of colony-stimulating factor 1 receptor by PLX3397 prevents amyloid beta pathology and rescues dopaminergic signaling in aging 5xFAD mice. Int J Mol Sci. 2020;21:5553. 32756440 10.3390/ijms21155553 PMC7432084

[84] Sosna J, Philipp S, Albay R, Reyes-Ruiz JM, Baglietto-Vargas D, LaFerla FM, et al. Early long-term administration of the CSF1R inhibitor PLX3397 ablates microglia and reduces accumulation of intraneuronal amyloid, neuritic plaque deposition and pre-fibrillar oligomers in 5XFAD mouse model of Alzheimer’s disease. Mol Neurodegener. 2018;13:11. 29490706 10.1186/s13024-018-0244-x PMC5831225

[85] Spangenberg E, Severson PL, Hohsfield LA, Crapser J, Zhang J, Burton EA, et al. Sustained microglial depletion with CSF1R inhibitor impairs parenchymal plaque development in an Alzheimer’s disease model. Nat Commun. 2019;10:3758. 31434879 10.1038/s41467-019-11674-z PMC6704256

[86] Spangenberg EE, Lee RJ, Najafi Allison R, Rice RA, Elmore MRP, Blurton-Jones M, West BL, et al. Eliminating microglia in Alzheimer’s mice prevents neuronal loss without modulating amyloid-β pathology. Brain. 2016;139(Pt 4):1265–81. 26921617 10.1093/brain/aww016 PMC5006229

[87] Zhao R, Hu W, Tsai J, Li W, Gan W-B. Microglia limit the expansion of β-amyloid plaques in a mouse model of Alzheimer’s disease. Mol Neurodegener. 2017;12:47. 28606182 10.1186/s13024-017-0188-6 PMC5468952

[88] Kiani Shabestari S, Morabito S, Danhash EP, McQuade A, Sanchez JR, Miyoshi E, et al. Absence of microglia promotes diverse pathologies and early lethality in Alzheimer’s disease mice. Cell Rep. 2022;39: 110961. 35705056 10.1016/j.celrep.2022.110961 PMC9285116

[89] Sheedy FJ, Grebe A, Rayner KJ, Kalantari P, Ramkhelawon B, Carpenter SB, Becker CE, et al. CD36 coordinates NLRP3 inflammasome activation by facilitating intracellular nucleation of soluble ligands into particulate ligands in sterile inflammation. Nat Immunol. 2013;14:812–20. 23812099 10.1038/ni.2639 PMC3720827

[90] Bassil R, Shields K, Granger K, Zein I, Ng S, Chih B. Improved modeling of human AD with an automated culturing platform for iPSC neurons, astrocytes and microglia. Nat Commun. 2021;12:5220. 34471104 10.1038/s41467-021-25344-6 PMC8410795

[91] d’Errico P, Ziegler-Waldkirch S, Aires V, Hoffmann P, Mezö C, Erny D, et al. Microglia contribute to the propagation of Aβ into unaffected brain tissue. Nat Neurosci. 2022;25:20–5. 34811521 10.1038/s41593-021-00951-0 PMC8737330

[92] Kaji S, Berghoff SA, Spieth L, Schlaphoff L, Sasmita AO, Vitale S, et al. Apolipoprotein E aggregation in microglia initiates Alzheimer’s disease pathology by seeding β-amyloidosis. Immunity. 2024;57:2651. 39419029 10.1016/j.immuni.2024.09.014

[93] Venegas C, Kumar S, Franklin BS, Dierkes T, Brinkschulte R, Tejera D, et al. Microglia-derived ASC specks cross-seed amyloid-β in Alzheimer’s disease. Nature. 2017;552:355–61. 29293211 10.1038/nature25158

[94] Iliff JJ, Wang M, Liao Y, Plogg BA, Peng W, Gundersen GA, et al. A paravascular pathway facilitates CSF flow through the brain parenchyma and the clearance of interstitial solutes, including amyloid β. Sci Transl Med. 2012;4:147ra111. 22896675 10.1126/scitranslmed.3003748 PMC3551275

[95] Wyss-Coray T, Lin C, Yan F, Yu GQ, Rohde M, McConlogue L, et al. TGF-beta1 promotes microglial amyloid-beta clearance and reduces plaque burden in transgenic mice. Nat Med. 2001;7:612–8. 11329064 10.1038/87945

[96] Liao F, Zhang TJ, Jiang H, Lefton KB, Robinson GO, Vassar R, et al. Murine versus human apolipoprotein E4: differential facilitation of and co-localization in cerebral amyloid angiopathy and amyloid plaques in APP transgenic mouse models. Acta Neuropathol Commun. 2015;3:70. 26556230 10.1186/s40478-015-0250-y PMC4641345

[97] Wojtas AM, Kang SS, Olley Benjamin M, Gatherer M, Shinohara M, Lozano PA, Liu C-C, et al. Loss of clusterin shifts amyloid deposition to the cerebrovasculature via disruption of perivascular drainage pathways. Proc Natl Acad Sci U S A. 2017;114:E6962–71. 28701379 10.1073/pnas.1701137114 PMC5565413

[98] Feng W, Zhang Y, Wang Z, Xu H, Wu T, Marshall C, et al. Microglia prevent beta-amyloid plaque formation in the early stage of an Alzheimer’s disease mouse model with suppression of glymphatic clearance. Alzheimers Res Ther. 2020;12:125. 33008458 10.1186/s13195-020-00688-1 PMC7532614

[99] Sperling RA, Jack CR, Black SE, Frosch MP, Greenberg SM, Hyman BT, et al. Amyloid-related imaging abnormalities in amyloid-modifying therapeutic trials: recommendations from the Alzheimer’s Association Research Roundtable Workgroup. Alzheimers Dement. 2011;7:367–85. 21784348 10.1016/j.jalz.2011.05.2351 PMC3693547

[100] Sperling R, Salloway S, Brooks DJ, Tampieri D, Barakos J, Fox NC, et al. Amyloid-related imaging abnormalities in patients with Alzheimer’s disease treated with bapineuzumab: a retrospective analysis. Lancet Neurol. 2012;11:241–9. 22305802 10.1016/S1474-4422(12)70015-7 PMC4063417

[101] Taylor X, Noristani HN, Fitzgerald GJ, Oluoch H, Babb N, McGathey T, et al. Amyloid-β (Aβ) immunotherapy induced microhemorrhages are linked to vascular inflammation and cerebrovascular damage in a mouse model of Alzheimer’s disease. Mol Neurodegener. 2024;19:77. 39434125 10.1186/s13024-024-00758-0 PMC11494988

[102] Condello C, Yuan P, Schain A, Grutzendler J. Microglia constitute a barrier that prevents neurotoxic protofibrillar Aβ42 hotspots around plaques. Nat Commun. 2015;6:6176. 25630253 10.1038/ncomms7176 PMC4311408

[103] Zeng H, Huang J, Zhou H, Meilandt WJ, Dejanovic B, Zhou Y, et al. Integrative in situ mapping of single-cell transcriptional states and tissue histopathology in a mouse model of Alzheimer’s disease. Nat Neurosci. 2023;26:430–46. 36732642 10.1038/s41593-022-01251-x PMC11332722

[104] Mallach A, Zielonka M, van Lieshout V, An Y, Khoo JH, Vanheusden M, et al. Microglia-astrocyte crosstalk in the amyloid plaque niche of an Alzheimer’s disease mouse model, as revealed by spatial transcriptomics. Cell Rep. 2024;43: 114216. 38819990 10.1016/j.celrep.2024.114216

[105] Mathys H, Boix CA, Akay LA, Xia Z, Davila-Velderrain J, Ng AP, et al. Single-cell multiregion dissection of Alzheimer’s disease. Nature. 2024;632:858–68. 39048816 10.1038/s41586-024-07606-7 PMC11338834

[106] Miyoshi E, Morabito S, Henningfield CM, Das S, Rahimzadeh N, Shabestari SK, et al. Spatial and single-nucleus transcriptomic analysis of genetic and sporadic forms of Alzheimer’s disease. Nat Genet. 2024;56:2704–17. 39578645 10.1038/s41588-024-01961-x PMC11631771

